# Saam Acupuncture for Treating Functional Dyspepsia: A Feasibility Randomized Controlled Trial

**DOI:** 10.1155/2022/2581041

**Published:** 2022-06-29

**Authors:** Boram Lee, O.-Jin Kwon, Joo-Hee Kim, Jung Won Kang, Tae-Hun Kim, Seunghoon Lee, Jungeun Kim, Ae-Ran Kim, So Young Jung, Hyo-Ju Park, Sun-Mi Choi

**Affiliations:** ^1^KM Science Research Division, Korea Institute of Oriental Medicine, Daejeon, Republic of Korea; ^2^Department of Acupuncture and Moxibustion Medicine, College of Korean Medicine, Sangji University, Wonju, Gangwon-do, Republic of Korea; ^3^Research Institute of Korean Medicine, Sangji University, Gangwon-do, Republic of Korea; ^4^Department of Acupuncture and Moxibustion, College of Korean Medicine, Kyung Hee University, Seoul, Republic of Korea; ^5^Korean Medicine Clinical Trial Center, Korean Medicine Hospital, Kyung Hee University, Seoul, Republic of Korea; ^6^Acupuncture, Moxibustion & Meridian Research Group, Korea Institute of Oriental Medicine, Daejeon, Republic of Korea; ^7^Clinical Research Coordinating Team, Korea Institute of Oriental Medicine, Yuseong-daero 1672, Yuseong-gu, Daejeon, Republic of Korea; ^8^KM Data Division, Korea Institute of Oriental Medicine, Daejeon, Republic of Korea; ^9^Korean Convergence Medical Science, University of Science & Technology (UST), Daejeon, Republic of Korea

## Abstract

**Objective:**

Functional dyspepsia (FD) is a common gastrointestinal disorder that significantly affects sufferers' quality of life and increases the economic burden on society. Saam acupuncture, a form of traditional Korean acupuncture, is frequently used to treat FD in Korean medicine clinical settings. This study aimed to evaluate the feasibility and preliminary effectiveness and safety of Saam acupuncture for treating FD.

**Methods:**

We conducted a pilot, pragmatic, assessor-blinded randomized controlled trial. Patients with FD according to the ROME III criteria were randomly allocated to an acupuncture plus usual care group or a usual care group. Saam acupuncture based on individualized FD and systemic symptoms was conducted in the acupuncture group three times per week for 4 weeks. Study feasibility outcomes, including recruitment, completion, and acupuncture adherence rates, were calculated. In addition, preliminary evaluation of participant responses to the intervention was tested using the gastrointestinal symptom (GIS), FD-related quality of life (FD-QoL), visual analog scale (VAS), patient global assessment (PGA), and EuroQol-5 Dimensions (EQ-5D) scores.

**Results:**

Twenty-four participants who met the eligibility criteria were included. The recruitment and completion rates of the clinical trials were 60% and 79.2%, and the acupuncture adherence rate was 83.3%. Although there was no significant difference between the two groups in the dyspepsia symptoms as measured by GIS, VAS, and PGA at Week 4, significant differences were found between the two groups at the follow-up assessments (Weeks 8 and 12). In particular, the early satiety subscore of GIS was significantly improved in the Saam acupuncture group compared with the usual care group at Week 4. The quality of life measured by FD-QoL and EQ-5D improved only in the Saam acupuncture group, although there were no significant differences between the two groups. No adverse events related to Saam acupuncture were reported.

**Conclusions:**

Saam acupuncture can be a feasible, preliminarily effective, and safe treatment for FD. Further confirmatory trials with a larger sample size are needed to confirm its effectiveness and safety. The trail is registered with CRIS-KCT0000164, URL: https://cris.nih.go.kr/cris/search/detailSearch.do/2098.

## 1. Introduction

Functional dyspepsia (FD) refers to the presence of one or more symptoms of epigastric pain, epigastric burning, early satiation, and postprandial fullness without organic, metabolic, or systemic disease. The symptoms are severe enough to interfere with usual activities over the last 3 months after an onset of at least 6 months prior [1]. The prevalence is reported to be very high worldwide, 10–21%, causing a global burden [2, 3]. It is not a life-threatening disease, but it reduces sufferers' quality of life with chronic and repetitive symptoms. The direct and indirect costs of FD to patients have been estimated to be $18.4 billion for the US population, imposing a significant economic burden on the healthcare system [4].

According to the American College of Gastroenterology and the Canadian Association of Gastroenterology guidelines on dyspepsia, *Helicobacter pylori* eradication, proton pump inhibitors (PPIs), tricyclic antidepressants, and prokinetic therapy are recommended for patients with FD [5]. However, because of the heterogeneous and diverse characteristics of FD symptoms [6] and the possible side effects of conventional medication, including extrapyramidal reactions [7], almost 50% of patients were found to use complementary and integrative medicine (CIM) such as acupuncture and herbal medicine [8].

Acupuncture is one of the most popular CIM treatments, and it is used for the treatment and management of various diseases. According to the data provided by the Health Insurance Review & Assessment Service of South Korea in 2020, FD is a common disease that ranks in the top 8 in terms of the number of patients visiting Korean medicine (KM) clinics [9], and acupuncture is actively used to treat FD in clinical settings.

Saam (Sa-Ahm) acupuncture is a unique acupuncture method of traditional KM, believed to have been developed in the mid-17th century by a Korean Buddhist monk named “Saam.” It differs from other Chinese acupuncture methods in that it uses the five shu points, located on the distal upper and lower limbs, belonging to the self-meridian and other meridians according to the cold-heat and deficiency-excess pattern identification [10, 11]. Therefore, the therapeutic purpose of Saam acupuncture is to control the balance between the five elements and the cold-heat and deficiency-excess pattern balance between the viscera and bowels [12]. According to surveys conducted among KM clinical doctors, acupuncture is used most often on FD patients in clinical settings, with body and Saam acupuncture used most frequently [13]. However, although studies related to acupuncture for the treatment of FD are being actively conducted, mainly in China [14–16], there have been no clinical trials on the actual effect of Saam acupuncture, which has been used in the KM clinical field on FD. Therefore, we aimed to generate basic evidence regarding the preliminary effectiveness and safety of Saam acupuncture on FD patients and to evaluate the feasibility of a confirmatory clinical trial.

## 2. Methods

### 2.1. Study Design

This trial was a pilot, pragmatic, assessor-blinded randomized controlled trial that examined the feasibility and preliminary effectiveness and safety of Saam acupuncture for treating FD patients. A total of 24 patients were randomly assigned to a 4-week acupuncture plus usual care group or a usual care group. It was conducted from March 2011 to March 2013 at Daejeon KM Hospital of Daejeon University in the Republic of Korea in accordance with the Declaration of Helsinki. Ethical approval was obtained from the institutional review board (approval number: djomc-55). In addition, the protocol was registered in the web database of the Clinical Research Information Service (registration number: KCT0000164). The trial was designed and reported following the Consolidated Standards of Reporting Trials (CONSORT) and the Standards for Reporting Interventions in Clinical Trials of Acupuncture (STRICTA) recommendations [17, 18].

### 2.2. Participants

Among those who agreed to participate in the trial and voluntarily signed an informed consent form after listening to a clear explanation of the purpose and characteristics of the clinical trial, those who met the following conditions were included: (1) those aged between 20 and 65, (2) those who were diagnosed with FD according to the ROME III criteria [19] (having at least one symptom among the following: postprandial fullness, early satiety, epigastric burning, and epigastric pain for the past 3 months with symptom onset at least 6 months before the screening visit, without any evidence of structural disease that was likely to explain the symptoms on upper gastrointestinal endoscopy), and (3) those with a dyspepsia symptom severity score of at least 40 points on a 0–100 visual analog scale (VAS) during the past week at the time of the screening visit.

Participants who met the following conditions were excluded: (1) those who were at risk of gastrointestinal motility stimulus due to gastrointestinal bleeding, mechanical intestinal obstruction, or perforation; (2) those having alarm symptoms such as severe weight loss, melena, dysphagia, repeated vomiting, anemia, jaundice, abdominal mass, or ascites; (3) those having received KM treatment for the treatment of FD during the last 4 weeks; (4) those having undergone a gastrointestinal surgery except for appendectomy in the past or those who had a disease such as cholangitis or pancreatitis; (5) those having been judged not appropriate for participating in this clinical trial due to severe cardiovascular disease, severe neurological disease, acute or chronic liver disease such as liver cancer or liver cirrhosis, malignant tumor, chronic lung and respiratory disease, poorly controlled hypertension or diabetes, drug addiction, alcoholism, chronic renal failure, active *tuberculosis*, other infectious diseases, mental disease, or a diet disorder that occurred during the last year; (6) those having taken medications that could affect the results of the trial as a conventional FD treatment or by causing indigestion as side effects, including PPIs, H2-receptor antagonists, corticosteroids, and nonsteroidal anti-inflammatory drugs (NSAIDs), within the last 1 month as self-reported and confirmed prescription; (7) those having hemorrhagic disease or taking anticoagulant drugs (except for aspirin); (8) those having experienced a hypersensitivity reaction after a previous acupuncture treatment; (9) those being held in group facilities such as social welfare facilities; (10) women who were pregnant or lactating or planned to become pregnant during the trial period; (11) those having participated in other clinical trials during the past 3 months; and (12) those judged inappropriate for participating in this trial by the investigators.

### 2.3. Randomization and Blinding

An independent statistician generated a random sequence number using SAS version 9.1.3 software (SAS Institute Inc., Cary, NC, USA), with an allocation ratio of 1 : 1 to each group. A block randomization method was used with a block size of 4, without stratification. Opaque envelopes with random assignment codes were sealed and stored in a double-locked cabinet. For the participants who met the inclusion criteria and did not meet the exclusion criteria, the investigators completed all evaluations at the baseline visit and then opened the opaque sealed envelopes in order in front of the participants and assigned them to each group.

Although blinding was not possible for the clinicians conducting the acupuncture treatment or the study participants due to the nature of the study design, the outcome assessor and data analyst were blinded to the group allocation during the trial period.

### 2.4. Intervention

When the inclusion and exclusion criteria were reviewed at the screening visit, and it was judged by the investigators that participation in the clinical trial was appropriate, the participants were enrolled in this clinical trial within 10 days of the screening visit and randomly assigned to the acupuncture plus usual care group or the usual care group.

In the acupuncture group, individualized Saam acupuncture treatment was performed, based on its use in the KM clinical field [11–13], with reference to the consensus among five KM specialists, textbooks on acupuncture and moxibustion [20], and external expert advice. Saam acupuncture, based on The Classic of Difficult Issues (Nanjing), was combined with the engendering and restraining relationship between the five elements. A total of four acupuncture points located in distal limbs were prescribed by selecting two acupuncture points from the self and other meridians, respectively, according to the cold-heat and deficiency-excess pattern [21] (Supplement 1). Individualized Saam acupuncture consisting of four acupuncture points was conducted based on the judgment of a KM specialist with more than 5 years of clinical experience and more than 6 years of regular medical education, considering the FD symptoms and systemic symptoms that each patient complained of, according to the principles of a pragmatic clinical trial. In accordance with the principles of the Saam acupuncture method, acupuncture was performed on the left side of men and the right side of women.

Disposable sterile acupuncture needles (0.25 × 30 mm; DongBang Acupuncture Inc., Seoul, Republic of Korea) were inserted into each acupuncture point at a depth of 0.2–1.5 cun and maintained for 20 min. The manipulation technique was applied until the local “de qi” sensation (soreness, numbness, heaviness, and distention) was achieved. Acupuncture treatment was performed three times a week for 4 weeks, a total of 12 times, and according to the principles of pragmatic clinical trials, the acupuncture therapist was allowed to communicate with the participants about the daily management and treatment of symptoms during the treatment period. Participants who did not receive three or more consecutive acupuncture treatments or received fewer than 10 out of 12 treatments in the acupuncture group were dropped out.

Except for the KM treatment, all kinds of treatment for FD, such as over-the-counter drugs and prescription drugs, were allowed, and all concomitant treatments were recorded in a case report form (CRF). However, treatments for symptoms other than FD that could affect dyspepsia symptoms, such as long-term NSAID use for arthritis, were prohibited during the clinical trial period under the judgment of the investigators. Education on FD lifestyle management was conducted using a brochure. The usual care group received the same treatment as the acupuncture group except the acupuncture treatment.

### 2.5. Outcome Measures

The primary outcome measure was feasibility outcomes to examine the feasibility of a confirmatory clinical trial, including recruitment rate (percentage of the number of enrolled participants to the total number of screening participants), completion rate (percentage of the number of participants who completed the clinical trial without dropping out to the total number of enrolled participants), attrition rate, and adherence rate of acupuncture (percentage of participants, after excluding those who did not receive three or more consecutive acupuncture treatments or who received fewer than 10 out of 12 treatments, in the acupuncture group).

The preliminary evaluation of participant response to the intervention was tested using gastrointestinal symptom (GIS), FD-related quality of life (FD-QoL), VAS, 3-level version of the EuroQol-5 Dimensions (EQ-5D-3L), and patient global assessment (PGA) scores at Weeks 4, 8, and 12.

The GIS questionnaire is a validated, self-rating 10-item questionnaire assessing dyspepsia-specific symptoms. The 10 items include nausea, vomiting, bloating, abdominal cramps, early satiety, acidic eructation/heartburn, sickness, loss of appetite, retrosternal discomfort, and epigastric/upper abdominal pain and are evaluated on a 5-point Likert scale (0–4) [22, 23], with a higher score indicating more severe indigestion. The GIS scores were measured at Weeks 0 (baseline visit), 4 (treatment termination), 8 (follow-up after final intervention), and 12 (second follow-up after final intervention).

With 21 items, the FD-QoL questionnaire assesses four dimensions of patients, including psychological status, role-functional status, eating status, and liveliness status [24]. Each item is evaluated on a 5-point Likert scale (0–4), with a higher score indicating lower quality of life. In addition, the VAS with a measurement range of 0 (no discomfort) to 100 (most painful condition) was performed to evaluate the overall discomfort caused by FD. The average daily symptoms for the last week were investigated. The EQ-5D-3L assesses health-related quality of life, including mobility, personal care, usual activities, pain/discomfort, and anxiety/depression [25, 26]. We used a validated Korean EQ-5D-3L questionnaire and calculated the quality-adjusted life year from the score. The PGA was used to evaluate how well the patients' FD symptoms improved compared to before treatment. The patients responded to the overall improvement after treatment using a 5-point scale, ranging from “much improved” to “much worse.”

In addition, we evaluated the safety of the intervention by investigating adverse events (AEs) that occurred during the study period through medical examinations and the participants' self-reports. The severity and causal relationships between the AEs and the intervention were recorded in the CRFs.

### 2.6. Sample Size and Statistical Analysis

This study was the first preliminary clinical trial to evaluate the effectiveness, safety, and study feasibility of Saam acupuncture for FD patients. Due to a lack of previous studies, we could not find adequate references for a sample size calculation, and therefore, this preliminary study was undertaken to provide a reference for this calculation. According to a study that recommended a minimum of 12 participants per group for a pilot study [27], we aimed to recruit a total of 24 participants, with 12 participants per group for assessing feasibility.

A statistician (OJK) independent of the intervention and evaluation conducted all statistical analyses using SAS version 9.4 software (SAS Institute Inc.). An intention-to-treat analysis method was used that included all participants who received acupuncture treatment and an assessment at least once after randomization. After checking the satisfaction of the normality assumption, a two-sided test with a significance level of 0.05 was performed using an analysis of covariance with the baseline as the covariate and the treatment group as the fixed factor. In addition, the differences before and after the treatment within each group were verified by a paired *t*-test. Multiple imputations were applied for missing values. A repeated-measures analysis of variance was performed to test the difference in the trend change by visits between each group.

## 3. Results

### 3.1. Study Participants

**Figure 1:**
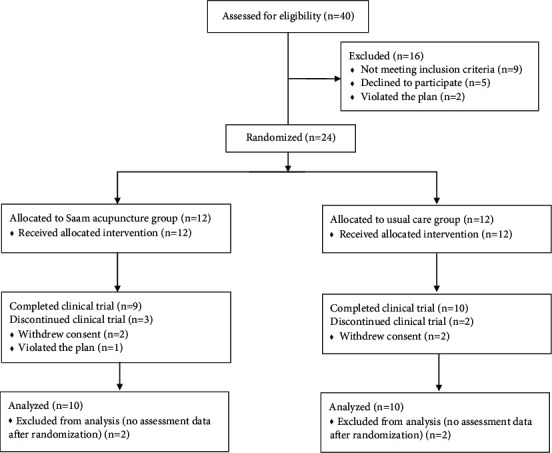
Study flowchart.

A total of 40 patients were assessed for eligibility. Among them, nine patients who did not meet the inclusion and exclusion criteria, five patients who declined to participate, and two patients who violated the protocol because they did not visit within 10 days of the screening visit were excluded. Twenty-four participants were randomly assigned 1 : 1 to the Saam acupuncture plus usual care group or the usual care group (Figure 1). There were no significant differences in baseline demographic characteristics between the two groups, including sex, age, and medical history (Table 1). There was no significant difference in eating habits between the two groups. The participants regularly ate at least twice a day and consumed stimulating foods (salty or spicy foods) or flour-based foods at most two to three times a week. Two participants in the acupuncture group and three participants in the usual care group took conventional medications such as acid suppressants and prokinetics during the study period.

### 3.2. Feasibility

The recruitment rate was 60% (24/40) in this trial. A total of five participants (20.8%; three participants in the acupuncture group and two participants in the control group) dropped out after randomization. Among them, four participants (two participants in the acupuncture and control groups, resp.) withdrew their consent during the intervention period, and one participant in the acupuncture group was excluded due to him/her violating the study protocol by not attending the last follow-up visit (Week 12). There were no dropouts due to AEs. Therefore, the assessment of the final outcomes was completed for a total of 19 participants (nine participants in the acupuncture group and ten participants in the control group) at the last follow-up visit (Week 12). The completion rates in the acupuncture and control groups were 75% (9/12) and 83.3% (10/12), respectively (total, 79.2%). In the acupuncture group, the adherence rate was 83.3% (10/12). It took 21 months at one center to recruit all 24 participants (1.14 participants recruited per center per month), and there were no missing data collection values, except for those due to participants' dropout.

### 3.3. Effectiveness

**Figure 2:**
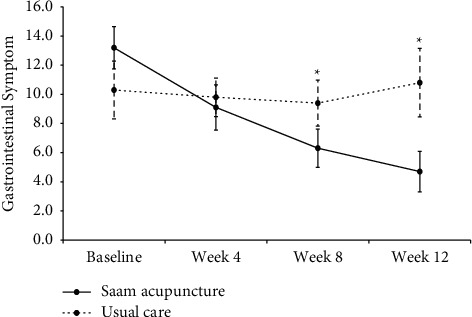
Mean gastrointestinal symptom scores during the study period. ^*∗*^Significant difference between the two groups, *P* < 0.05.

Although there was no significant difference between the two groups in total GIS scores at Week 4 (*P*=0.3050), significant differences were found between the two groups at the follow-up assessments (Weeks 8 and 12; *P*=0.0339 and 0.0113). After applying a repeated-measures analysis of variance, the GIS scores of the usual care group showed little change, while those of the acupuncture group continued to decrease after treatment (Figure 2). Among the 10 subitems of the GIS, the early satiety scores showed significant differences between the two groups during the treatment period, especially during Week 4 (*P*=0.0098). In addition, there were significant differences between the two groups at Weeks 8 and 12 for the bloating scores (*P*=0.0238 and 0.0083) and at Week 8 for the sickness (*P*=0.0225) and loss of appetite scores (*P*=0.0102). The scores for epigastric pain, one of the main symptoms of FD, were significantly improved at Weeks 8 and 12 (*P*=0.0205 and 0.0091) compared to the baseline scores only in the acupuncture group, although there was no significant difference between the two groups (Table 2).

The FD-QoL scores were significantly improved compared to baseline only in the acupuncture group, but there was no significant difference between the two groups. However, among the FD-QoL subitems, there was a significant difference between the two groups for eating status at Week 12 (*P*=0.0039). The scores for the VAS, which measured the intensity of dyspepsia symptoms, significantly improved during the treatment period in the acupuncture group, and there was a significant difference between the two groups at Weeks 8 and 12 (*P*=0.0069 and 0.0021). The EQ-5D-3L scores were significantly improved compared to baseline at Week 8 (*P*=0.0387) only in the acupuncture group, but there was no significant difference between the two groups (Table 3).

**Figure 3:**
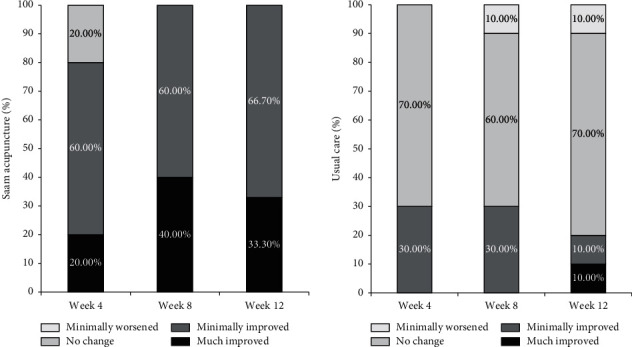
Results of patient global assessment during the study period.

In the acupuncture group at Week 4, two participants (20%) described their condition as “much improved,” six (60%) as “minimally improved,” and two (20%) as “no change” compared to before treatment. In the usual care group, three (30%) described their condition as “minimally improved” and seven (70%) as “no change” compared to before treatment. At Week 8, four participants (40%) described their condition as “much improved” and six (60%) as “minimally improved” compared to before treatment in the acupuncture group, while three (30%) described their condition as “minimally improved,” six (60%) as “no change,” and one (10%) as “minimally worse” in the usual care group. At Week 12, three participants (33.3%) described their condition as “much improved” and six (66.7%) as “minimally improved” compared to before treatment in the acupuncture group, while one (10%) described their condition as “much improved,” one (10%) as “minimally improved,” seven (70%) as “no change,” and one (10%) as “minimally worse” in the usual care group. Although there was no significant difference in the PGA scores between the groups at Week 4 (*P*=0.1025), there were statistically significant differences at Weeks 8 and 12 (*P*=0.0026 and 0.0025) (Figure 3).

### 3.4. Safety

During the clinical trial, one serious AE occurred in the usual care group due to hospitalization for a urinary stone. The symptoms were cured after extracorporeal shock wave lithotripsy, and it was judged to clearly not be related to acupuncture because it occurred in the usual care group.

## 4. Discussion

In this preliminary clinical trial assessing the feasibility of confirmatory research, the recruitment rate was 60% (24/40), the completion rate was 79.2% (19/24), and the adherence rate of acupuncture was 83.3% (10/12). These results suggest that the compliance and study feasibility of this clinical trial were relatively high, given that adherence to long-term therapy for chronic illnesses in developed countries is typically 50% [28]. In our study, a mean of 1.14 participants was recruited per month in one center. This is slightly higher than trials funded and published by the UK's National Institute for Health Research, which recruited a median of 0.92 participants per center per month [29]. There were no missing values during data collection, except for those due to participant dropout, indicating that the participants understood and completed the questionnaire and other data collection measures. This indicates the preliminary feasibility of the data collection procedure.

In addition, we confirmed that Saam acupuncture improved dyspepsia as measured by GIS, VAS, and PGA and health-related quality of life as measured by FD-QoL and EQ-5D-3L. Although there was no significant difference in GIS scores between the two groups at Week 4, the difference increased over time, and there was a significant difference between them when measured at Weeks 8 and 12 for the follow-up assessments.

In our study, FD participants who met the ROME III criteria were recruited, but the ROME IV criteria are currently being applied. However, there is no significant change in the diagnostic criteria for FD between these two criteria, so the results of this study are applicable when applying the current criteria. According to the current ROME criteria, FD is divided into postprandial distress syndrome (PDS) with early satiety and postprandial fullness as the main symptoms and epigastric pain syndrome (EPS) with epigastric pain and burning as the main symptoms. Based on the prevalence studies of the existing FD subdivision, it has been reported that the prevalence of PDS is twice as high as that of EPS worldwide [30–33]. In our study, interestingly, Saam acupuncture significantly improved the early satiety subscores of the GIS compared to the control group at all evaluation points. This may suggest the possibility of Saam acupuncture for improving PDS. We aimed to evaluate the feasibility and preliminary effectiveness of Saam acupuncture in a small sample of FD patients, and all included participants had PDS symptoms. That is, there were no patients with only EPS symptoms without PDS symptoms in our study. Therefore, it would be meaningful to confirm the effect of Saam acupuncture according to the FD subdivisions in a future larger trial.

The quality of life measured by FD-QoL and EQ-5D-3L was only improved in the acupuncture group. It has been suggested that most diseases are caused by interruptions or imbalances in the meridian network [34]. The theory of Saam acupuncture is based on the regulation and harmonization of Qi and the blood among the organs and meridians, and it simultaneously modulates relative channels, which are selected based on the theory of nourishing or suppressing cycle relationships, to ensure whole-body balance [35]. Therefore, Saam acupuncture might have improved systemic function, and it might have led to a decrease in the FD-QoL's liveliness status, psychological status, and role-functioning status scores and the EQ-5D-3L total scores in addition to the severity of the dyspeptic symptoms. However, there was no statistically significant difference between the two groups in terms of these outcomes, so these results should be interpreted carefully.

In our study, Saam acupuncture treatment was performed by a KM specialist with more than 5 years of clinical experience and more than 6 years of regular medical education. No AEs occurred during the trial period, and therefore, we could confirm the preliminary safety of Saam acupuncture. These findings are consistent with previous studies indicating that acupuncture is relatively safe when performed by a qualified acupuncturist [36]. In addition, since the Saam acupuncture method stimulates acupuncture points at the ends of the limbs, there is practically no risk of internal organ damage.

Saam acupuncture is frequently used for FD treatment in the KM clinical field, and we assessed its effect through a pragmatic clinical trial design, selecting Saam acupuncture points based on FD symptoms and the systemic symptoms of each patient. Through this, we aimed to increase the external validity of the study results by reducing the gap between the real-world clinical setting and the research. In addition, according to the existing studies, Saam acupuncture as an adjunctive or alternative therapy to body acupuncture improves back pain and sciatica or stroke patient's dysarthria symptoms compared with body acupuncture alone [37, 38]. In situations where new treatment alternatives for FD are required due to the heterogeneous characteristics of FD and the side effects of conventional medications [6, 7], we confirmed the preliminary effect, safety, and potential of Saam acupuncture for treating FD through this study.

Various mechanisms have been proposed for acupuncture in patients with FD. In particular, a recent systematic review revealed that acupuncture improved dyspepsia by enhancing gastric motility and accommodation, regulating gastrointestinal hormones and mental status, and improving central and autonomic functions [39]. However, as far as we know, the specific mechanism of Saam acupuncture in FD treatment has not been studied. The acupuncture points of Saam acupuncture are located at the ends of the four limbs. Therefore, it would be interesting to conduct a mechanistic study of how Saam acupuncture stimulation of the extremities affects the upper gastrointestinal tract. Additional studies should be conducted in the future to elucidate the mechanism of how Saam acupuncture affects this heterogeneous disease.

Our research has the following limitations. Since we did not set sham acupuncture as a control group, we could not evaluate the preliminary efficacy of Saam acupuncture, which is a major limitation of our study. The purpose of this study was to explore the preliminary effectiveness, safety, and feasibility of Saam acupuncture for treating FD patients, and usual care was set as the control group to reflect real-world conditions. Therefore, blinding of the practitioner and participants was not possible. However, we attempted to minimize the risk of potential bias through blinding of the outcome assessors. In addition, according to the characteristics of a pragmatic clinical trial, we reflected the real-world clinical setting by allowing communication between participants and the acupuncture therapists during treatment in the acupuncture group. However, nonspecific effects in this context may have affected the results of the trial. Furthermore, the feasibility outcome of our study was not initially planned at the time of protocol development. However, we have reported this outcome to provide relevant information for performing future clinical trials.

Through our study, to the best of our knowledge, we verified for the first time the preliminary effectiveness and safety of Saam acupuncture, which is widely used in KM clinical settings for FD patients, using a validated assessment questionnaire. In addition, we assessed the feasibility of conducting large confirmatory clinical trials. Based on the results of our study, conclusive evidence of the efficacy of Saam acupuncture in FD patients should be obtained through long-term, confirmatory clinical trials with formal sample size calculations.

## 5. Conclusions

Our study suggested the potential feasibility, preliminary effectiveness, and safety of Saam acupuncture for FD. Further high-quality confirmatory trials with a larger sample size and long-term follow-up are needed to demonstrate the exact effectiveness and safety of Saam acupuncture.

## Figures and Tables

**Table 1:** Baseline characteristics of the included participants.

Characteristics^a^	Saam acupuncture (*N* = 10)	Usual care (*N* = 10)	*P* value
Gender (male/female)^b^	2 (20.0%)/8 (80.0%)	4 (40.0%)/6 (60.0%)	0.6285
Age (year)^c^	47.90 (40.70, 55.10)	49.90 (40.44, 59.36)	0.7079
BMI (kg/m^2^)^c^	21.22 (19.28, 23.16)	21.75 (20.52, 22.97)	0.6117
Exercise (yes/no)^b^	3 (30.0%)/7 (70.0%)	8 (80.0%)/2 (20.0%)	0.0698
Smoking (yes/no)^b^	0 (0.0%)/10 (100.0%)	1 (10.0%)/9 (90.0%)	0.9999
Drinking (yes/no)^b^	2 (20.0%)/8 (80.0%)	5 (50.0%)/5 (50.0%)	0.3498
Caffeine (yes/no)^b^	5 (50.0%)/5 (50.0%)	4 (40.0%)/6 (60.0%)	0.9999
Medical history (yes/no)^b^			
Conventional medication	7 (70.0%)/3 (30.0%)	4 (40.0%)/6 (60.0%)	0.3698
Korean medicine	5 (50.0%)/5 (50.0%)	4 (40.0%)/6 (60.0%)	0.9999
Functional dyspepsia subtype^b^			
PDS	3 (30.0%)	3 (30.0%)	0.9999
PDS + EPS	7 (70.0%)	7 (70.0%)	

*Notes*. ^a^Data are presented as mean and 95% confidence interval or number (%). ^b^Fisher's exact test. ^c^Independent *t*-test. BMI: body mass index; EPS: epigastric pain syndrome; PDS: postprandial distress syndrome.

**Table 2:** Gastrointestinal symptom score.

Variables	Saam acupuncture (*N* = 10)		Usual care (*N* = 10)		*P* value (between-group)
	Mean (95% CI)	*P* value (within-group)	Mean (95% CI)	*P* value (within-group)	
GIS (total)					
Baseline	13.20 (9.60, 16.80)		10.30 (5.60, 15.00)		
Week 4	9.10 (5.23, 12.97)	0.0523	9.80 (6.66, 12.94)	0.7263	0.3050
Week 8	6.30 (3.03, 9.57)	**0.0046**	9.40 (5.64, 13.16)	0.5228	**0.0339**
Week 12	4.70 (1.26, 8.14)	**0.0071**	10.80 (5.22, 16.38)	0.7919	**0.0113**
GIS (nausea)					
Baseline	0.60 (0.00, 1.29)		0.50 (0.00, 1.01)		
Week 4	0.60 (0.00, 1.29)	0.9999	0.40 (0.03, 0.77)	0.6783	0.6148
Week 8	0.50 (0.00, 1.11)	0.7263	0.60 (0.00, 1.20)	0.7577	0.7045
Week 12	0.40 (0.00, 0.90)	0.4433	0.80 (0.14, 1.46)	0.4344	0.3066
GIS (vomiting)					
Baseline	0.30 (0.00, 0.65)		0.30 (0.00, 0.65)		
Week 4	0.40 (0.00, 1.09)	0.7263	0.10 (0.00, 0.33)	0.1679	0.3363
Week 8	0.10 (0.00, 0.33)	0.3434	0.10 (0.00, 0.33)	0.3434	0.9999
Week 12	0.10 (0.00, 0.33)	0.1679	0.70 (0.02, 1.38)	0.2695	0.1000
GIS (bloating)					
Baseline	2.10 (1.06, 3.14)		2.10 (1.24, 2.96)		
Week 4	1.70 (0.80, 2.60)	0.4790	1.90 (1.49, 2.31)	0.6193	0.6546
Week 8	0.80 (0.24, 1.36)	**0.0063**	1.80 (0.99, 2.61)	0.4961	**0.0238**
Week 12	0.90 (0.11, 1.69)	**0.0368**	2.20 (1.39, 3.01)	0.8402	**0.0083**
GIS (abdominal cramps)					
Baseline	0.80 (0.24, 1.36)		0.80 (0.00, 1.61)		
Week 4	0.70 (0.11, 1.29)	0.5911	0.20 (0.00, 0.50)	0.0811	0.0551
Week 8	0.70 (0.11, 1.29)	0.6783	0.40 (0.00, 0.90)	0.3434	0.3814
Week 12	0.20 (0.00, 0.50)	0.0510	0.90 (0.00, 1.82)	0.7804	0.1120
GIS (early satiety)					
Baseline	2.20 (1.26, 3.14)		1.90 (0.98, 2.82)		
Week 4	1.20 (0.75, 1.65)	**0.0085**	1.80 (1.14, 2.46)	0.7263	**0.0098**
Week 8	1.10 (0.57, 1.63)	**0.0115**	1.80 (1.24, 2.36)	0.7263	**0.0077**
Week 12	0.70 (0.11, 1.29)	**0.0017**	1.70 (1.02, 2.38)	0.5086	**0.0003**
GIS (acidic eructation)					
Baseline	1.30 (0.62, 1.98)		1.20 (0.46, 1.94)		
Week 4	1.10 (0.31, 1.89)	0.5086	1.00 (0.25, 1.75)	0.6618	0.9854
Week 8	0.80 (0.00, 1.68)	0.0957	1.00 (0.33, 1.67)	0.6926	0.6381
Week 12	0.60 (0.00, 1.29)	0.0662	0.80 (0.06, 1.54)	0.2229	0.7103
GIS (sickness)					
Baseline	0.80 (0.06, 1.54)		0.40 (0.00, 0.90)		
Week 4	0.50 (0.00, 1.11)	0.1934	0.70 (0.02, 1.38)	0.0811	0.0662
Week 8	0.30 (0.00, 0.65)	0.0522	0.60 (0.23, 0.97)	0.3434	**0.0225**
Week 12	0.30 (0.00, 0.78)	0.2443	1.00 (0.25, 1.75)	0.1114	0.1066
GIS (loss of appetite)					
Baseline	1.70 (0.58, 2.82)		1.30 (0.34, 2.26)		
Week 4	1.00 (0.42, 1.58)	0.2569	1.30 (0.54, 2.06)	0.9999	0.3867
Week 8	0.60 (0.10, 1.10)	**0.0243**	1.30 (0.71, 1.89)	0.9999	**0.0102**
Week 12	0.60 (0.00, 1.20)	0.0751	1.10 (0.31, 1.89)	0.4433	0.1481
GIS (retrosternal discomfort)					
Baseline	1.30 (0.34, 2.26)		0.80 (0.14, 1.46)		
Week 4	0.80 (0.24, 1.36)	0.3809	1.10 (0.06, 2.14)	0.2789	0.3852
Week 8	0.70 (0.11, 1.29)	0.1934	0.80 (0.00, 1.80)	0.9999	0.4403
Week 12	0.30 (0.00, 0.65)	0.0629	0.80 (0.14, 1.46)	0.9999	0.1231
GIS (epigastric pain)					
Baseline	2.10 (1.24, 2.96)		1.00 (0.33, 1.67)		
Week 4	1.10 (0.24, 1.96)	0.0957	1.30 (0.71, 1.89)	0.0811	0.3334
Week 8	0.70 (0.11, 1.29)	**0.0205**	1.00 (0.33, 1.67)	0.9999	0.3113
Week 12	0.60 (0.10, 1.10)	**0.0091**	0.80 (0.06, 1.54)	0.4433	0.3165

*Notes*. Bold values mean statistically significant differences (*P* < 0.05). CI: confidence interval; GIS: gastrointestinal symptom.

**Table 3:** Functional dyspepsia-related quality of life, numeric rating scale, and the 3-level version of EuroQol-5 Dimensions scores.

Variables	Saam acupuncture (*N* = 10)		Usual care (*N* = 10)		*P* value (between-group)
	Mean (95% CI)	*P* value (within-group)	Mean (95% CI)	*P* value (within-group)	
FD-QoL (total)					
Baseline	38.50 (27.37, 49.63)		31.50 (17.24, 45.76)		
Week 4	28.30 (18.16, 38.44)	0.0131	26.80 (12.30, 41.30)	0.2956	0.4824
Week 8	19.00 (9.69, 28.31)	0.0034	24.50 (10.01, 38.99)	0.1688	0.1432
Week 12	18.30 (9.34, 27.26)	0.0016	24.10 (8.70, 39.50)	0.1418	0.1126
FD-QoL (eating status)					
Baseline	11.70 (8.28, 15.12)		7.70 (3.30, 12.10)		
Week 4	9.10 (6.25, 11.95)	0.0730	7.10 (−3.45, 10.75)	0.5599	0.7309
Week 8	6.10 (2.81, 9.39)	0.0113	6.30 (2.37,10.23)	0.3217	0.2839
Week 12	4.70 (2.46, 6.94)	0.0007	7.30 (2.85, 11.75)	0.7509	0.0039
FD-QoL (liveliness status)					
Baseline	9.80 (6.81, 12.79)		8.00 (5.48, 10.52)		
Week 4	6.10 (3.68, 8.52)	0.0392	7.20 (4.49, 9.91)	0.5628	0.3635
Week 8	4.80 (2.87, 6.73)	0.0046	6.50 (3.63, 9.37)	0.2637	0.1456
Week 12	4.60 (3.33, 5.87)	0.0035	5.40 (2.34, 8.46)	0.0719	0.3773
FD-QoL (psychological status)					
Baseline	8.90 (4.51, 13.29)		7.70 (3.20, 12.20)		
Week 4	6.70 (3.02, 10.38)	0.0864	6.20 (1.78, 10.62)	0.2932	0.8280
Week 8	4.00 (0.87, 7.13)	0.0186	5.50 (1.33, 9.67)	0.2481	0.3232
Week 12	4.00 (0.53, 7.47)	0.0113	5.60 (1.36, 9.84)	0.2681	0.2959
FD-QoL (role-functioning status)					
Baseline	8.10 (4.08, 12.12)		8.10 (3.31, 12.89)		
Week 4	6.40 (2.64, 10.16)	0.1816	6.30 (1.25, 11.35)	0.1737	0.9528
Week 8	4.10 (2.18, 6.02)	0.0343	6.20 (1.64, 10.76)	0.1915	0.2286
Week 12	5.00 (2.08, 7.92)	0.0792	5.80 (1.38, 10.22)	0.0984	0.7261
VAS					
Baseline	68.10 (58.08, 78.12)		62.30 (54.28, 70.32)		
Week 4	46.80 (31.72, 61.88)	0.0184	55.50 (43.17, 67.83)	0.3681	0.3539
Week 8	27.30 (9.69, 44.91)	0.0012	57.20 (45.88, 68.52)	0.4303	0.0069
Week 12	33.80 (18.72, 48.88)	0.0017	62.90 (48.52, 77.28)	0.9249	0.0021
EQ-5D-3L					
Baseline	0.836 (0.750, 0.922)		0.841 (0.770, 0.913)		
Week 4	0.848 (0.780, 0.916)	0.5862	0.867 (0.812, 0.921)	0.4121	0.5892
Week 8	0.894 (0.844, 0.945)	0.0387	0.855 (0.762, 0.947)	0.7198	0.2776
Week 12	0.888 (0.832, 0.944)	0.0521	0.828 (0.760, 0.897)	0.7061	0.0855

*Notes*. CI: confidence interval; EQ-5D-3L: the 3-level version of EuroQol-5 Dimensions; FD-QoL: functional dyspepsia-related quality of life; VAS: visual analog scale.

## Data Availability

The data that support the findings of this study are available from the corresponding author (Sun-Mi Choi) upon reasonable request, as are the individual deidentified participant data.

## Supplementary Materials

Supplementary Materials Supplement 1. Saam's combination of five Shu points for deficiency, excess, cold, and heat symptoms of the meridians.
