# Supplementary material for: Saam Acupuncture for Treating Functional Dyspepsia: A Feasibility Randomized Controlled Trial
Source: Evid Based Complement Alternat Med. 2022 Jun 29;2022:2581041. doi: 10.1155/2022/2581041 (PMC9259230; doi:10.1155/2022/2581041)
Supplement: Supplementary Materials — Supplement 1. Saam's combination of five Shu points for deficiency, excess, cold, and heat symptoms of the meridians. [file 2581041.f1.docx]

Supplement 1. Saam's combination of five Shu points for deficiency, excess, cold, and heat symptoms of the meridians

| Meridian | Deficiency | | | | Excess | | | | Cold | | | | Fire | | | |
| --- | --- | --- | --- | --- | --- | --- | --- | --- | --- | --- | --- | --- | --- | --- | --- | --- |
|  | Tonify | | Sedate | | Tonify | | Sedate | | Tonify | | Sedate | | Tonify | | Sedate | |
| Lung | SP3 | LU9 | HT8 | LU10 | HT8 | LU19 | KI10 | LU5 | HT8 | LU10 | LU5 | KI10 | LU5 | KI10 | SP3 | LU9 |
| Large intestine | ST36 | LI11 | SI5 | LI5 | SI5 | LI5 | BL66 | LI2 | SI5 | ST41 | LI2 | BL66 | LI2 | BL66 | SI5 | ST41 |
| Stomach | SI5 | ST41 | GB41 | ST43 | GB41 | ST43 | LI1 | ST45 | ST41 | SI5 | ST44 | BL66 | ST44 | BL66 | ST36 | BL54 |
| Spleen | HT8 | SP2 | LR1 | SP1 | LR1 | SP1 | LU8 | SP5 | SP2 | HT8 | SP9 | KI10 | SP9 | KI10 | SP3 | KI3 |
| Heart | LR1 | HT9 | KI19 | HT3 | KI19 | HT3 | SP3 | HT7 | HT8 | KI2 | HT3 | KI10 | HT3 | KI10 | HT8 | KI2 |
| Small intestine | GB41 | SI3 | BL66 | SI2 | BL66 | SI2 | ST36 | SI8 | SI5 | BL60 | SI2 | BL66 | SI2 | BL66 | SI8 | ST36 |
| Bladder | LI1 | BL67 | ST36 | BL54 | ST36 | BL54 | GB41 | BL65 | SI5 | BL60 | SI2 | BL66 | SI2 | BL66 | ST36 | BL54 |
| Kidney | LU8 | KI7 | SP3 | KI3 | SP3 | KI3 | LR1 | KI1 | HT8 | KI2 | KI10 | HT3 | KI10 | HT3 | SP3 | KI3 |
| Pericardium | LR1 | PC9 | KI10 | PC3 | KI10 | PC3 | SP3 | PC7 | HT8 | PC8 | PC3 | HT3 | PC3 | HT3 | SP3 | PC7 |
| Triple energizer | GB41 | TE3 | BL66 | TE2 | BL66 | TE2 | ST36 | TE10 | TE6 | BL60 | TE2 | BL66 | TE2 | BL66 | TE6 | BL60 |
| Gall bladder | BL66 | GB43 | LI1 | GB44 | LI1 | GB44 | SI5 | GB38 | GB38 | SI5 | GB43 | BL66 | GB43 | BL66 | BL54 | GB34 |
| Liver | KI10 | LR8 | LU8 | LR4 | LU8 | LR4 | HT8 | LR2 | LR2 | HT8 | KI10 | LR8 | KI10 | LR8 | LR3 | SP3 |
